# Outbreak of pertussis at community A in Dormaa Municipality, Ghana, August 2016

**DOI:** 10.11604/pamj.supp.2018.30.1.15290

**Published:** 2018-05-18

**Authors:** Florence Nzilanye Iddrisah, Samuel Dapaah, Meeyoung Mattie Park, Daniel Owusu-amponsah, Joseph Asamoah Frimpong, Scott JN McNabb, Ernest Kenu, Edwin Andrew Afari, Ernest Konadu Asiedu

**Affiliations:** 1 Ghana Field Epidemiology and Laboratory Training Program, Accra, Ghana; 2 Rollins School of Public Health, Emory University, Atlanta, USA; 3 Ghana Health service, Ghana; 4 African Field Epidemiology Network, Accra, Ghana

**Keywords:** Outbreak investigation, pertussis, Ghana

## Abstract

Pertussis is a vaccine preventable disease (VPD) monitored by the World Health Organization (WHO). Despite a long-established Pertussis immunization system, the re-emergence of the disease in some countries stressed the need to have well-trained field epidemiologists at the forefront in the fight against these VPDs, especially during an outbreak. Practical, hands-on training is useful for clearer understanding of the principles and development of competencies relevant to outbreak investigation, which will enhance field practice; case method training using realistic public health scenarios helps trainees put into practice learned theory. As such, this case study was adopted from a real Pertussis outbreak investigation that was conducted by Ghana’s Field Epidemiology Training Program residents, together with the rapid response team members of Dormaa Municipal health directorate in August 2016. It was primarily designed for training novice public health practitioners in a facilitated classroom setting. Participants should be able to complete the exercises in approximately 3 hours.

## How to use this Study

**General instructions:** ideally, 1 to 2 instructors facilitate the case study for 8 to 20 students in a classroom or conference room. The instructor should direct participants to read a paragraph out loud, going around the room to give each participant a chance to read. When the participant reads a question, the instructor directs all participants to perform calculations, construct graphs, or engage in discussions. The instructor may split the class to play different roles or take different sides in answering a question. As a result, participants learn from each other, not just from the instructors. Specific instructor’s notes are included with each question in the instructor’s version of this case study.

**Audience:** residents in Frontline Field Epidemiology Training Programs (FETP-Frontline), Field Epidemiology and Laboratory Training Programs (FELTPs), and others who are interested in this topic.

**Prerequisites:** before using this case study, case study participants should have received lectures or other instruction in outbreak investigation.

**Materials needed:** laptop with Microsoft Excel or graph paper, flipchart or white board with markers.

Level of training and associated public health activity: Novice - Outbreak investigation

**Time required:** approximately 3 hours

**Language:** English

## Competing interest

The authors declare no competing interest.
